# CRISPR-dCas9-Based Artificial Transcription Factors to Improve Efficacy of Cancer Treatment With Drug Repurposing: Proposal for Future Research

**DOI:** 10.3389/fonc.2020.604948

**Published:** 2021-02-03

**Authors:** Alejandro Martinez-Escobar, Benjamín Luna-Callejas, Eva Ramón-Gallegos

**Affiliations:** Environmental Cytopathology Laboratory, Department of Morphology, Escuela Nacional de Ciencias Biológicas, Instituto Politécnico Nacional, Mexico City, Mexico

**Keywords:** cancer treatment, drug repurposing, CRISPR-Cas9, artificial transcription factors, CRISPR-dCas9-based ATFs

## Abstract

Due to the high resistance that cancer has shown to conventional therapies, it is difficult to treat this disease, particularly in advanced stages. In recent decades, treatments have been improved, being more specific according to the characteristics of the tumor, becoming more effective, less toxic, and invasive. Cancer can be treated by the combination of surgery, radiation therapy, and/or drug administration, but therapies based on anticancer drugs are the main cancer treatment. Cancer drug development requires long-time preclinical and clinical studies and is not cost-effective. Drug repurposing is an alternative for cancer therapies development since it is faster, safer, easier, cheaper, and repurposed drugs do not have serious side effects. However, cancer is a complex, heterogeneous, and highly dynamic disease with multiple evolving molecular constituents. This tumor heterogeneity causes several resistance mechanisms in cancer therapies, mainly the target mutation. The CRISPR-dCas9-based artificial transcription factors (ATFs) could be used in cancer therapy due to their possibility to manipulate DNA to modify target genes, activate tumor suppressor genes, silence oncogenes, and tumor resistance mechanisms for targeted therapy. In addition, drug repurposing combined with the use of CRISPR-dCas9-based ATFs could be an alternative cancer treatment to reduce cancer mortality. The aim of this review is to describe the potential of the repurposed drugs combined with CRISPR-dCas9-based ATFs to improve the efficacy of cancer treatment, discussing the possible advantages and disadvantages.

## Introduction

Cancer represents one of the most important health challenges in the world. It can be treated by the combination of surgery, radiation therapy, and/or drug administration. Surgery and radiation are used to treat cancer that is confined locally and drug therapy is used to kill metastasized cancer cells (1, 2). Cancer therapies provide different efficiency degrees depending on the tumor type and therapy applied; however, anticancer drug-based therapies are the main treatment used in different tumor types (2). Anticancer drugs are classified as pregenomic and genomic era drugs. Pregenomic era drugs are targeted against a tumor phenotype, whereas genomic era drugs are developed after the target is identified by using molecular techniques that consider intratumoral genetic heterogeneity (1, 3, 4). Furthermore, cancer drug development takes an average of 11.4–13.5 years and an investment from 161 to 1,800 million dollars per drug (5–7). An alternative solution to this problem is drug repurposing which is the application of a drug for another indication than it was originally approved. It helps to reduce development costs and gets a more rapid return on investment in the development of repurposed drugs (7–11). Some repurposed drugs have demonstrated antitumor efficacy by inducing cancer cell death or suppressing various genes related to cancer (12). There are different mechanisms by which the repurposed drugs cause antitumor effects, however, it is important to study mechanisms that regulate gene expression related to proliferation and cell death to improve the cancer treatment efficacy and avoid drug resistance. The possibility to combine pregenomic era drugs and molecular tools could increase tumor cell killing and reduce the likelihood of drug resistance (1).

The artificial transcription factors (ATFs) are molecular tools that can manage the gene expression to induce changes in different cell stages (13, 14). Within the different types of ATFs, the emerging CRISPR-dCas9-based ATFs have been used to precisely regulate gene expression in different *in vitro* and *in vivo* studies. Aforementioned, these molecular tools are a promising strategy for cancer treatment at the transcriptional level (15, 16). In addition, it is important in cancer research to identify new drug combinations that generate synergistic effects and thereby achieve more efficient therapies (4, 17).

For this reason, in this review, we described the possibility to implement a cancer therapy with CRISPR-dCas9-based ATFs combined with repurposed drugs, to regulate gene expression related to pharmacodynamics of the repurposed drug and/or MDR genes of the cancer cells.

## Drug Repurposing Used in Cancer Therapies

Cancer drug development requires preclinical and clinical studies to extensively test and characterize their pharmacological properties, efficacy, antineoplastic effects, and toxicity (5, 12). The time to develop and license new drugs are often longer than the identification of new targets for chemotherapeutic intervention (18). The pharmacodynamics of the cancer drug has to be identified and validated to proceed to clinical trials. For that reason, drug repurposing is a great opportunity for alternative cancer therapy development, since it is faster, safer, easier, and cheaper (3) and because most of the non-cancer drugs have little or tolerable adverse effects for human health, contrary to chemotherapeutic agents that have relevant side effects that significantly reduce life quality (19).

Despite drug repurposing in cancer advantages, drugs are affected by multidrug-resistant (MDR) mechanisms that decrease their pharmacodynamic, enhance degradation of the drug, and reduce uptake. In this way, it is important to tackle genetic heterogeneity and drug resistance in cancer through the drug combination with molecular tools. One possible solution for this situation is to use CRISPR technology to silence MDR genes and increase cancer treatment effectiveness (1).

## CRISPR Therapy in Chemosensitivity

Multiple drug resistance is caused by the differential expression of genes in tumor cells, commonly called multidrug resistance genes (MDR). This resistance is responsible for unsuccessful chemotherapies and causing high mortality in a short time. An alternative to overcome this challenge is to silence or inactivate these MDR genes (20–22). In recent years, the clustered, regularly interspaced short palindromic repeats (CRISPR) in combination with a CRISPR-associated nuclease 9 (Cas9) have been used for this purpose due to its practical use, versatility, and its cleavage efficiency in almost any target sequence (20, 23). The CRISPR-Cas9 system is formed of an RNA-guided endonuclease (Cas9/sgRNA complex) which consists of the single guided RNA (sgRNA) fuses with Cas9. The sgRNA is formed by a CRISPR RNA (crRNA) and a trans-activating crRNA (tracrRNA) (21, 22).

For cancer therapy, each CRISPR therapeutic target is selected by the tumor type. For example, CRISPR-Cas9 targeting the CXC chemokine receptor 4 (CXCR4) was evaluated *in vitro* and *in vivo* studies on hepatocarcinoma, which significantly decreased its expression and inhibited cell proliferation and migration leading to less invasiveness and also significantly increased the chemosensitivity to cisplatin (24). In another study, the CRISPR-Cas9 system was used to deactivate the Nuclear Erythroid 2-Related Factor (NRF2) gene in lung cancer cells. It showed an increase in the sensitivity to chemotherapeutic agents such as cisplatin and carboplatin (25).

Similarly, CRISPR has been evaluated to increase chemosensitivity in breast cancer by inactivating or down-regulating the MDR1 gene (also known as ABCB1) that significantly increased the doxorubicin cytotoxicity in resistant chemotherapy breast cancer cells. These data suggested that the mutation of the MDR1 gene by intracellular administration of the CRISPR-Cas9 complex recovered the drug susceptibility and avoided multidrug resistance in breast cancer cells (26).

In ovarian cancer, chemosensitivity with CRISPR-Cas9 has also been increased from the inactivation of the MDR1 gene that encodes the P-gp protein. This decrease in expression was associated with a greater sensitivity to doxorubicin (27). Likewise, the PARP-1 gene has been suppressed by CRISPR-Cas9 in ovarian cancer and caused a greater sensitivity to cisplatin in cancer cells (28). In osteosarcoma, P-gp expression can be effectively blocked by CRISPR-Cas9, and P-gp inhibition was associated with reversal of doxorubicin resistance in MDR osteosarcoma cell lines (KHOSR2 and U-2OSR2). For that reason, the CRISPR-Cas9 system increased the long-term chemotherapy efficacy by overcoming P-gp-mediated MDR in the clinical setting (29).

Although sometimes it is enough to inactivate a gene to reverse chemotherapy resistance, the tumor types can have several target genes that can lead to the same goal of making it chemosensitive. For example, p53 was overexpressed to make cells more sensitive to doxorubicin chemotherapy and a greater effect on chemosensitization of resistant osteosarcoma cells was obtained (30).

Due to the above, the use of the CRISPR-Cas9 tool combined with chemotherapy can enhance the efficacy of the elimination of various tumor cell types. However, the specificity of Cas9/sgRNA needs to be carefully evaluated since Cas9/sgRNA can have undesired off-target targets and cut essential genes for the patient (20). For this reason, the CRISPR-dCas9 system has been used due to its deactivated nickase activity that does not make cuts in the genetic sequence and it does not permanently inactivate genes and thereby reduces desire off-target effects (31, 32).

## CRISPR-dCas9-Based Artificial Transcription Factors

**Figure 1:**
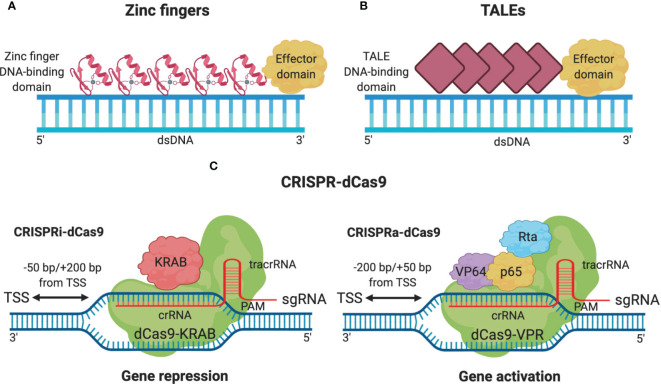
ATFs types used in the repression or activation of transcription. **(A)** Zinc finger-based ATF is composed of an effector domain and a DNA-binding domain like the Cys2-His2 (C2H2) domain which contains multiple cysteine and histidine residues which are ligands for the zinc ion. **(B)** TALEs-based ATF also consists of an effector domain and a DNA-binding domain (diamond red) composed of 33–35 amino acid repeat arrays (each repeat domain specifies a single DNA base). **(C)** CRISPR-dCas9-based ATFs comprise a dCas9 protein and a single guide RNA (sgRNA). Interference of transcription mediated by dCas9 associated with KRAB domain (CRISPRi) and activation of transcription associated with VP64, p65, and Rta domains that conglomerate ribonucleic complexes (RNAP) to activate the transcription process (CRISPRa). Created with BioRender.com.

ATFs are used to express and/or suppress target genes. They consist of molecular domains such as DNA-binding domains (DBD) that confer sequence specificity and may target similar sites in the genome with different affinity degrees. Several DBDs are used for the design of ATFs, including zinc fingers (ZF) (**Figure 1A**), transcription activator-like effectors (TALEs) (**Figure 1B**), and the CRISPR-dCas9 system (**Figure 1C**) (14, 32, 33).

ATFs also have an effector domain (ED) that interact with DBD to activate or repress transcription of target genes by blocking the transcription process (14, 34, 35).

The CRISPR-dCas9 system nuclease activity is deactivated by mutations (Cas9 mutated is called dCas9) and an ED can be incorporated to allow its function as ATFs. The dCas9 retained the DNA-binding specificity of wild-type Cas9, without causing a DNA double-strand cleavage altering the host DNA sequence (26). Furthermore, the dCas9 protein requires sgRNA for its specificity. Multiple sgRNAs can be easily designed and synthesized, making the CRISPR-dCas9 system suitable for testing more than one target simultaneously (14, 35–37).

The combinatorial effect of the use of CRISPR-dCas9-based ATFs with certain chemotherapeutics makes it possible to completely eradicate tumor cells. It has been seen that a long non-coding RNA (lncRNA) KCNQ1OT1 was overexpressed in squamous cell carcinoma tissues and lung cancer which were resistant to cisplatin (38, 39). By using CRISPR-dCas9-based ATFs (**Figure 1C**) with interfering function in expression called CRISPR interference (CRISPRi), KCNQ1OT1 expression was inhibited in CAL27-res and SCC9-res cells that improved the chemosensitivity to cisplatin. While using CRISPR-dCas9-based ATFs with activating function in expression called CRISPR activator (CRISPRa), expression levels of KCNQ1OT1 increased by promoting cell growth and returning chemoresistance in the cells. The CRISPR-dCas9-based ATFs are useful tools in gene overexpression and underexpression, which improves chemosensitivity (38).

Currently, CRISPR-dCas9-based ATFs have been applied in drug resistance, epigenetic regulation, and immune regulation in various cell lines such as squamous cell carcinoma (CAL27-res and SCC9-res), breast cancer (E0771), pancreatic adenocarcinoma (Pan02), melanoma cells (B16F10), hepatoma (Hep3B), lung (H157), etc (22, 38, 40–42).

CRISPRa has been used to express the target antigenic peptide (SIINFEKL) in breast cancer (E0771), pancreatic adenocarcinoma (Pan02), and melanoma (B16F10) cells, in an orthotopic model in mice to enhance the elimination of tumor cells through the immune response generated by the peptide (40). Similarly, in lung cancer cells (H157), CRISPRa activated the expression of MASPIN (mammary serine protease inhibitor) that led to a concomitant cell proliferation inhibition and apoptosis induction (42).

Several *in vivo* studies with mice models have validated the use of CRISPR-dCas9-based ATFs as regulators of the gene expression related to cancer development. However, more studies on the off-target effects of this tool are still lacking before moving to clinical phases (36). In addition, CRISPR technologies *in vivo* transfection efficiencies are still relatively low; hence for the implementation of this technology in the clinic for cancer treatment, it is necessary to continue with scientific research on the most plausible *in vivo* administration of these ATFs to target tissues (43). The ideal *in vivo* delivery system should cause low immunogenicity and direct the dCas9/sgRNA to the interested organ or cell type (44). There is a variety of *in vivo* delivery systems like viral vectors (adenovirus and lentivirus) that are very efficient (36), but they could have side effects due to their potential carcinogenesis and immunogenicity (15, 45, 46). Another delivery system is the DNA plasmid. However, since the size of CRISPR/Cas9 plasmids is larger than other plasmids, they exhibit a higher charge density, and more polycations are required to condense them (46, 47). Currently, with the help of nanotechnology, different administration methods of the system based on metal, polymeric, or lipid nanoparticles have emerged (44, 48, 49). The use of these nanoparticles can improve transfection efficiency, reduce off-target effects, decrease systemic toxicity, and immune risks associated with transfection (45).

Since cancer is involved in multiple and complex cellular pathways that affect the efficacy of the therapies, drug combination therapies might be an alternative strategy to have a higher success rate in the clinical application (17). For this reason, in this review, it is proposed to use CRISPR-dCas9-based ATFs for cancer therapies in combination with repurposed drugs whose action mechanisms are to regulate the expression of oncogenes and tumor suppressor genes or to inactivate MDR genes. **Table 1** summarizes the repurposed drugs analyzed in this study from several pharmacological classes. As selection criteria, all drugs are currently being evaluated in clinical trials for cancer therapy, and they have been observed in *in vitro* studies on various cancer types, which have an action mechanism with antitumor effect due to their ability to regulate gene expression involved in cell proliferation and death (5, 12, 19). It is proposed to combine the gene regulation effects of repurposed drugs and the CRISPR-dCas9-based ATFs to obtain a cancer therapy with a higher success rate. With this combination, target genes can be synergistically regulated from CRISPR-dCas9-based ATFs to enhance the effect of repurposed drugs. Additionally, genes involved in the signaling pathway of processes related to cancer development can be complementarily regulated, as well as, MDR genes can be silenced to have a higher success rate in the treatment (4–6, 17, 19).

**Table 1:** Repurposed drugs proposed for cancer therapy combination with CRISPR-dCas9-based ATFs.

Drug	Pharmacological class	Original indication	Effect of the drug on gene regulation related to cancer	Complementary gene regulation with CRISPR-dCas9	Cancer types	Refs.
Digitoxin	Cardiac glycosides	Cardiac conditions	Expression of p21	Suppression of HIF-1 and HIF-2	Prostate, lung, breast	(12, 19, 50)
Chlorpromazine	Antipsychotic drugs	Psychosis, schi-zophrenia, bipolar disorder	Expression of p21 Suppression of oncogene K-Ras	Expression of p53	Colorectal, glioma, leukemia	(12, 51, 52)
Mebendazole	Microbiological agents	Parasitic worm infection	Expression of pro-apoptotic Bcl-2	Suppression of ABL and BRAF oncogenes	Colorectal, melanoma, glioblastoma	(12, 53–55)
Ritonavir	Antiviral	HIV treatment	Expression of p53 Suppression of pRb	Expression of p21	Ovary, breast pancreatic	(12, 56)
Nelfinavir	Antiviral	HIV treatment	Expression of DR5 and inhibition of AKT	Expression of SREBP-1 and ATF6	Lung, ovary, breast	(12, 57)
Naproxen	NSAIDs	Antiinflammatory	Suppression of genes for COX-enzymes	Expression of PI3K	Leukemia, breast, colorectal, osteosarcoma	(12, 58)
Ibuprofen	NSAIDs	Pain, fever, antiinflammatory	Expression of Akt, p53, Bcl-2 and Bax	Suppression of genes for COX-enzymes	Colorectal, melanoma	(12, 59, 60)
Aspirine	Salicylate	Pain, fever	Suppression of Sp transcription factors family	Suppression of genes for COX-enzymes	Colorectal	(4, 6, 19, 53–55)
Metformine	Oral antidiabetic	Diabetes II	Inhibition of mTORC1 and Activation of AMPK	Expression of AMPK	Hepatocarcinoma, breast, colorectal, prostate	(12, 56)
Artesunate	Microbiological agents	Malaria	Expression of pro-apoptotic proteins such as caspase-3	Suppression of antiapoptotic proteins and MYC oncogenes.	Lymphoma, myeloma, hepatocarcinoma	(12, 61, 62)
Itraconazole	Microbiological agents	Fungal infections	Inhibition of 14-alfa-lanosterol demethylase	Decreased AKT1 activity	Lung, prostate	(12, 63)
Doxycycline	Antibiotic	Bacterial infections	Suppression of MMP-2 and MMP-9	Expression of TIMP-2	Hepatocarcinoma, lung, prostate, colorectal	(12, 64, 65)
Lithium	Antidepressant	Depressant	Inhibition of glycogen synthase kinase 3	Suppression Smad3 and TGFBIp	Prostate, colorectal	(12, 66, 67)

## Discussion

Cancer is a complex, heterogeneous, and highly dynamic disease with multiple evolving molecular constituents. Due to the genomic instability of cancer cells, every individual cancer cell has a set of mutations. This tumor heterogeneity causes several resistance mechanisms in cancer therapy, mainly the target mutation (3, 4, 22). CRISPR-dCas9-based ATFs can be used in transcriptional therapeutics to optimize gene expression and design a more controllable system, for example, repurposed drug inducible system, improving the potency of gene manipulation, multiplexing and resource limitation and dosage and gene expression pattern (68). For cancer therapy, CRISPR-dCas9-based ATFs had been developed to activate tumor suppressor genes and silence oncogenes and the tumor resistance mechanisms for targeted therapy (22). Some potential strategies for CRISPR/Cas9 interventions targeting cellular genes in cancer have proposed downregulation of oncogenes (ErbB, *src, abl, fps, yes, ras, raf*, and *myc*) and genes related to chemoresistance (MDR-1, MRP, GST-p, UGT1A1 and Cytokine P450) and for upregulation of tumor suppressor genes (pRb, p53, APC, SMAD4, PTEN, BRCA1/2, and ATM) (22, 69).

For that reason, it is proposed to use CRISPR-dCas9-based ATFs for cancer therapies in combination with repurposed drugs whose action mechanisms are to regulate the expression of oncogenes and tumor suppressor genes or to inactivate MDR genes. This could allow for synergy or complementarity between CRISPR technology and repurposed drugs in cancer therapy since both strategies express or repress certain genes involved in cancer, and its combined use could generate a synergistic effect that enhances therapy when the repurposed drug regulates the expression of the same gene that will be the target for CRISPR-dCas9-based ATFs.

For example, in **Table 1**, the digitoxin causes cell cycle arrest in the G2/M-phase since it induces the expression of p21, an inhibitor of cyclin-dependent kinases and they suppress HIF-1 and HIF-2 expression which are transcription factors often increased in tumors that regulate essential genes related to hypoxic environments for tumor adaptation (12, 19, 50, 51). For this reason, the CRISPR-dCas9-based ATFs can be combined with these repurposed drugs to equally activate p21 expression and generate a synergistic effect or to suppress HIF family expression and generate a complementary effect to make chemotherapy more effective.

Cancer treatments are handled by multiple therapeutic tools. These can be used depending on the patient types and their diagnosis. In this sense, the repurposed drugs in combination with CRISPR-dCas9-based ATFs may be an innovative alternative that promises to be able to cover certain tumor types more efficiently. Drug repurposing and CRISPR-dCas9-based ATFs have been used for cancer therapy, and they have received increasing attention from biotechnology research due to the economic advantages they represent for the pharmaceutical industry, as well as the molecular advantages they confer on the patient during the cancer treatment. The use of drug repurposing alternatives for cancer treatment represents fewer side effects for patients and a wider range of applications as molecular advantages. Nevertheless, cancer efficacy of drug repurposing is still affected by MDR genes (1, 19). This challenge may be solved with CRISPR-Cas9 technology or CRISPR-dCas9-based ATFs in combination with drug repurposing by the inactivation of MDR genes. Despite CRISPR-Cas9 technology providing an effective inactivation of any gene, it cleaves one target at a time and in a non-specific way, which represents other disadvantages (25, 27, 29). For this reason, CRISPR-dCas9-based ATFs is the best option for cancer therapy combination since it not only has a majority of therapeutic targets but also the DNA double-strand is not broken, and the host DNA sequence is not altered. CRISPR-dCas9-based ATFs are even relatively cost-effective in comparison to the *de novo* construction of protein-based ZF and TALEs DNA-binding domains. Other advantages are that CRISPR-dCas9-based ATFs are more specific compared to TALEs and ZINC fingers and may have several genetic targets to regulate at the same time (36, 70). However, the CRISPR-dCas9 specificity can decrease depending on the complexity of the DNA due to the inaccessibility to the therapeutic target (43, 71).

Regarding CRISPR-dCas9-based ATFs limitations, the dCas9/sgRNA complex is bigger than other ATFs as TALEs or Zinc Fingers, and cell delivery may be difficult (14, 22, 35, 37).

Other limitations of the CRISPR-dCas9-based ATFs are the off-target effects due to the possibility of dCas9 binding to nucleotide sequences similar to the target PAM sequence. However, the optimization of the length of the sgRNA allows reducing the off-target effects without sacrificing efficiency in the objective (43, 72). Despite the above, more information is needed to corroborate the real negative impact of the off-target effects generated by dCas9 since it only performs partial and temporary binding with the off-target sequences without damaging them (36, 70).

Finally, cancer therapy with drug repurposing combined with CRISPR-dCas9-based ATFs has not yet been carried out on an experimental basis; however, it is important to explore in future research the possibility to combine these methods for cancer therapy due to the potential advantages to reduce cancer mortality in a cost-effective manner and with more efficient results.

## Author Contributions

AM-E and BL-C: Conceptualization, methodology, writing—original draft preparation. ER-G: Supervision, writing—reviewing, and editing. All authors contributed to the article and approved the submitted version.

## Funding

This project was financed by Secretaría de Investigación y Posgrado (SIP-IPN) through project 2020205 and Consejo Nacional de Ciencia y Tecnología (CONACyT) through Fondo Sectorial de Investigación para la Educación through project No. A1-S-21548.

## Conflict of Interest

The authors declare that the research was conducted in the absence of any commercial or financial relationships that could be construed as a potential conflict of interest.
